# Influences on Adherence to Antiretroviral Therapy (ART) in Early-Stage HIV Disease: Qualitative Study from Uganda and South Africa

**DOI:** 10.1007/s10461-020-02819-z

**Published:** 2020-09

**Authors:** Norma C. Ware, Monique A. Wyatt, Emily E. Pisarski, Bosco M. Bwana, Catherine Orrell, Stephen Asiimwe, Gideon Amanyire, Nicholas Musinguzi, David R. Bangsberg, Jessica E. Haberer

**Affiliations:** 1 Deparment of Medicine, Brigham and Women’s Hospital, Boston, MA, USA; 2 Deparment of Global Health and Social Medicine, Harvard Medical School, 641 Huntington Ave., Boston, MA 02115, USA; 3 Harvard Global, Cambridge, MA, USA; 4 Mbarara University of Science and Technology, Mbarara, Uganda; 5 Global Health Collaborative, Mbarara, Uganda; 6 Desmond Tutu HIV Foundation, Cape Town, South Africa; 7 University of Cape Town, Cape Town, South Africa; 8 Kabwohe Clinical Research Centre, Kabwohe, Uganda; 9 Makerere University Joint AIDS Program, Kampala, Uganda; 10 Oregon Health and Science University–Portland State University School of Public Health, Portland, OR, USA; 11 Massachusetts General Hospital Center for Global Health, Boston, MA, USA

**Keywords:** ART adherence, Early HIV disease, Qualitative study, Uganda, S. Africa

## Abstract

Realization of optimal treatment and prevention benefits in the era of universal antiretroviral therapy (ART) and “U=U” (undetectable = untransmittable) requires high adherence at all stages of HIV disease. This article draws upon qualitative interview data to characterize two types of influences on ART adherence for 100 Ugandans and South Africans initiating ART during early-stage HIV infection. Positive influences are: (a) behavioral strategies supporting adherence; (b) preserving health through adherence; (c) support from others; and (d) motivating effect of adherence monitoring. “De-stabilizing experiences” (mobility, loss, pregnancy) as barriers are posited to impact adherence indirectly through intervening consequences (e.g. exacerbation of poverty). Positive influences overlap substantially with adherence facilitators described for later-stage adherers in previous research. Adherence support strategies and interventions effective for persons initiating ART later in HIV disease are likely also to be helpful to individuals beginning treatment immediately upon confirmation of infection. De-stabilizing experiences merit additional investigation across varying populations.

## Introduction

Data demonstrating that viral suppression resulting from antiretroviral therapy (ART) greatly reduces HIV transmission [1–3] represented a major step forward in the effort to end the HIV epidemic. The ensuing “undetectable = untransmittable” (U= U) campaign has done much to disseminate new evidence that viral suppression in a person living with HIV all but eliminates infectiousness [4, 5]. World Health Organization guidelines now recommend that individuals with HIV begin treatment immediately upon confirmation of infection, regardless of stage of disease [6].

Eastern and southern Africa is the region of the world most affected by the HIV epidemic, accounting for 45% of new infections and 53% of persons living with HIV globally [7]. Uganda is one of the nations hardest hit by the epidemic. Initial HIV estimates revealed an 11% prevalence among pregnant women in Kampala in 1985; this estimate had risen to a high of 30% by 1992 [8]. Uganda is known for its “ABC” (Abstinence, Be Faithful, Use Condoms) approach to reducing the rate in HIV infection and succeeded in reversing the epidemic in-country in the 1990s [9–11]. UNAIDS estimates place the prevalence of HIV in Uganda in 2018 at 5.7%, meaning 1,400,000 Mio. people were living with HIV [12].

Both treatment and prevention benefits of ART are contingent upon high adherence. In sub-Saharan Africa, adherence has generally been high in advanced disease among those in care [13]. In Uganda specifically, early studies report rates of ART adherence in excess of 90% [14, 15]. However, data reporting adherence to early therapy, when symptoms are less prominent or entirely absent, are scarce.

A recently published systematic review and meta-analysis found that patients with higher (vs. lower) CD4 counts were less likely to achieve excellent adherence, although many studies found no difference between these groups. The authors of the meta-analysis called for additional high-quality studies, particularly among adults initiating ART at higher CD4 cell counts [16].

A recent prospective observational study of adherence in Ugandan and South African adults initiating ART in early vs. late-stage HIV disease (the Measuring Early Treatment Adherence, or “META” Study) tested the hypothesis that ART adherence may be lower in individuals with early-stage HIV infection. Adherence was monitored electronically for 12 months. Overall, results revealed adherence to be as high or higher (i.e. a greater proportion of expected device-openings observed) for early-stage ART initiators, with the exception of pregnant women in South Africa [17].

A qualitative study was conducted in conjunction with the larger META Study. The goal of the qualitative study was to identify and describe influences on adherence for early-stage ART initiators. Here we report results from the qualitative research.

## Methods

### The META Study

The META Study was carried out in rural southwest Uganda and in Gugulethu Township, near Cape Town, South Africa. Participants received ART and routine HIV care at local clinics. Three distinct groups of adults living with HIV were enrolled: (1) “early/non-pregnant”: men and non-pregnant women initiating ART with early-stage, asymptomatic infection (CD4 > 350 cells/μL); (2) “early/pregnant”: pregnant women initiating ART with early-stage, asymptomatic infection (CD4 > 350 cells/μL); and (3) “late/non-pregnant”: men and non-pregnant women initiating ART with late-stage infection (CD4 < 200 cells/μL). Participants were: (1) ≥ 18 years old; (2) ART-naïve and initiating ART within 1 month of study enrollment date; (3) living within 60 km of the clinic where they were enrolled and receiving HIV care; and (4) intending to stay in the area for 1 year. Pregnant women were ≤ 34 weeks in pregnancy according to the best available estimate. Nine-hundred-four (N = 904) individuals took part in the META Study.

ART adherence was assessed using a real-time electronic adherence monitor that transmits a date-and-time stamp over cellular networks each time the device is opened (Wisepill Technologies, South Africa). Participants were given monitoring devices at study enrollment, and asked to use the devices to store their ART. Research assistants (RAs) explained the purpose and function of the devices to participants, asking participants to store only their ART in the device and remove one dose at a time. If participants were hesitant to use the monitoring device (e.g. due to travel), they were advised to use an alternate pill container during those periods.

Adherence was calculated as the number of device openings observed divided by the number of openings expected over the 12-month follow-up period, adjusting for device malfunction (3% of follow-up). Percent adherence and sustained gaps in adherence were reported at follow-up Months 6 and 12.

A number of sociobehavioral factors potentially influencing adherence were investigated quantitatively via self-report on questionnaires administered at META Study follow-up visits. Among these was a questionnaire item asking respondents to rate their health on a five-point scale ranging from “poor” to “excellent”. Data from these health self-assessments informed sampling for the qualitative study, as described below.

### The Qualitative Study

#### Sampling and Recruitment

Purposeful sampling was used to select the qualitative sample from META Study participants. Because understanding adherence influences in early-stage HIV disease was the major focus of the qualitative work, participants were drawn exclusively from the “early/non-pregnant” and “early/pregnant” META Study groups. Purposeful sampling was used to maximize variation within each group, to set the stage for in-depth qualitative analysis. Early/non-pregnant qualitative participants included both men and women; early/pregnant participants included women who were pregnant and those who had recently given birth at the time of qualitative data collection. Wherever possible, we also purposefully identified individuals who characterized their health as good or excellent on the META Study health self-assessment, to increase the likelihood that participants would be asymptomatic and experiencing difficulties with adherence. Individuals receiving care at diverse clinical sites in both countries were included. Potential participants were referred to qualitative study RAs by META Study staff. RAs then approached them in person during META Study follow-up visits or via mobile phone to describe the qualitative research and extend an invitation to participate. Informed consent was obtained from all participants.

#### Data Collection

Qualitative study participants took part in a single, individual semi-structured interview conducted by a female, local qualitative study RA with bachelor’s level training. Interviews were carried out after participants had been enrolled in the META Study for at least 6 months. Interviews focused on experiences of taking daily ART, and prioritized the elicitation of personal “stories” on interview topics. Topics included: (a) HIV testing; (b) ART initiation; (c) perceptions of ART before beginning treatment and whether and how these had changed; (d) daily pill-taking routines; (e) missed doses and/or longer breaks from ART; (f) disclosure of HIV status; (g) relationships; (h) help (or absence of help) with adherence; (i) participation in the study, including using the electronic monitoring device; and (j) pregnancy (where applicable).

Interviews were carried out in quiet, private locations of participants’ choosing, in local languages (Runyankole, isiXhosa). Interviews were audio-recorded with consent, and lasted about 1 h. As soon as possible after each interview, content was transcribed directly into English by the RA who had conducted the interview.

#### Data Quality

Interview transcripts were reviewed regularly for data quality. Data quality feedback was delivered to qualitative study RAs through weekly calls and emails to improve interviewer technique and ensure that the topics covered in the interviews remained focused on study goals.

#### Analysis of Data

We used an inductive content analytic approach to analyze the qualitative data [18]. Interview transcripts were reviewed to identify sections of text suggestive of adherence influences. Each identified section of text was assigned a preliminary label. The labels served as the basis for development of a coding scheme. Data were coded by two coders according to the coding scheme using Atlas.ti qualitative data management software. Coders coded and compared a subset of transcripts, compared results, and resolved discrepancies through discussion until consistency in use of the codebook was reached. Also, half (N = 50) of the transcripts were summarized to capture personal stories on topics of major interest while preserving relevant context.

Descriptive categories were developed to represent adherence influences that were repeatedly and directly reported by interviewees (positive influences), or repeatedly suggested by the personal stories they told (“de-stabilizing experiences” as adherence barriers). Categories were developed by combining relevant content from the coded data, the summaries, and in some cases, the interview transcripts.

Category development consisted of: (1) writing summary descriptions of the concept represented by the category; (2) assigning a label; and (3) illustrating the concept with excerpts from the interview data. This approach to category development allows for transparency in the presentation of study results and enables readers to evaluate rigor by critically examining clarity, detail and internal consistency in category content. Descriptive categories resulting from the analysis are presented as “Results,” below.

## Results

### Participant Characteristics

One-hundred-three persons were enrolled in the qualitative study; three were not interviewed. One individual withdrew, one could not be reached to set up the interview appointment, and one was revealed to be ineligible following enrollment. Thus the final participant group numbered 100 individuals.

Selected personal characteristics of participants in the qualitative study and the larger META Study are presented in Table 1. From the table, we see that more than three-quarters (78%) of qualitative study participants were women; median age was 27. Slightly fewer than half (45%) reported being married, with an average of two children. Sixty-two percent rated their health at META Study baseline as “fair-to-good”; 34% considered themselves in “very good-to-excellent” health. Median CD4 (cells/mm^3^) at baseline was 446.5; more than 80% were virally suppressed at Month 12. Median adherence for qualitative participants was 88% at Month 6, and 81% at Month 12 of the META Study follow-up period.

Compared to the larger META Study sample, qualitative participants had higher CD4 counts (446.5 vs. 369 cells/mm^3^, p < 0.001) and better self-assessed health (very good/excellent health 34% vs. 15%, p < 0.001) at baseline. They were also younger (median age 27 vs. 31, p < 0.001). There were no significant differences in Month 6 or Month 12 adherence, or in viral suppression, between the two groups.

### Qualitative Results

Analysis of the qualitative data yielded two broad thematic categories characterizing influences on adherence for this study sample. They are entitled: (a) positive influences, and (b) de-stabilizing experiences. Positive influences are: (1) behavioral strategies to support adherence, (2) preserving health through adherence, (3) support from others, and (4) the motivating effect of adherence monitoring. De-stabilizing experiences are: (5) mobility, (6) loss, and (7) pregnancy. We propose de-stabilizing experiences negatively impact adherence indirectly, through their consequences. The impact of de-stabilizing experiences on adherence is addressed separately at the end of Qualitative Results, under the heading: de-stabilizing experiences and adherence. Data excerpts illustrating each category appear in Table 2 (positive influences) and Table 3 (de-stabilizing experiences).

### Positive Influences

#### Behavioral Strategies to Support Adherence

Qualitative study participants made use of behavioral strategies to support adherence. Prominent among these was reliance on reminders to cue pill-taking at the planned dosing time. Popular reminder strategies were setting alarms on watches or phones, and timing dosing to coincide with TV or radio programs. The adherence monitoring device also served as a reminder. Behavioral adherence strategies also included steps taken to ensure transport would be available on clinic appointment days (Table 2, 1).

#### Preserving Health Through Adherence

The desire for good health was a powerful motive to adhere. Despite initiating treatment in early-stage disease, many qualitative participants described seeking HIV testing because they felt unwell. Once they started taking ART, they experienced considerable symptom relief. Associating ART with feeling healthy became a reason to keep taking the pills.

Some were less concerned with feeling healthy than with preserving the appearance of good health. Not “looking sick” was considered a way of avoiding being identified as having HIV. In this way also, the association of ART adherence with good health fueled determination to take pills as prescribed.

For pregnant women, preserving the health of children was an important factor. Women described feeling a responsibility—as parents—to prevent their unborn children from becoming HIV-infected by taking ART as prescribed. Some also spoke in broader terms, referencing a need to remain healthy themselves so as to be able to care for and support their children until adulthood (Table 2, 2).

#### Support from Others

Participants reported receiving support from others for adherence. Support took the form of material resources, encouragement, and especially, reminders to take ART. Material resources might include money for transport to clinic, food, and/or help with household chores, and tended to be provided by family and friends. Encouragement was abundant—a frequent response to disclosure, then repeated at regular intervals. Supporters expressed encouragement by cautioning adherers to “keep taking their pills.” Reminders typically came from family members living in the same household, but more casual friends could also track dosing schedules closely, ensuring pills were taken on time.

Counseling, including specific counseling messages, was cited as an important source of support. Particularly in Uganda, participants described the comfort, reassurance, and sense of hope they derived from counseling received as part of HIV testing and ART initiation. Especially helpful was being told they were “not going to die” from HIV, and hearing they were “not alone” (Table 2, 3).

Support for adherence was widely referenced by qualitative study participants, yet some insisted they relied only on themselves to continue taking ART as prescribed. Generally, these individuals explained either that they “had no one” to help, or that they alone were responsible for their lives.

#### The Motivating Effect of Adherence Monitoring

The experience of adherence monitoring had multiple meanings for participants. Some saw the monitoring device as increasing disclosure risk by attracting attention; others felt it lowered disclosure risk because it wasn’t easily recognizable as a container for storing medication. A few interviewees described feeling “special” as a result of having the device. Some characterized the device as helping them remember to take their medication.

**Fig. 1:**
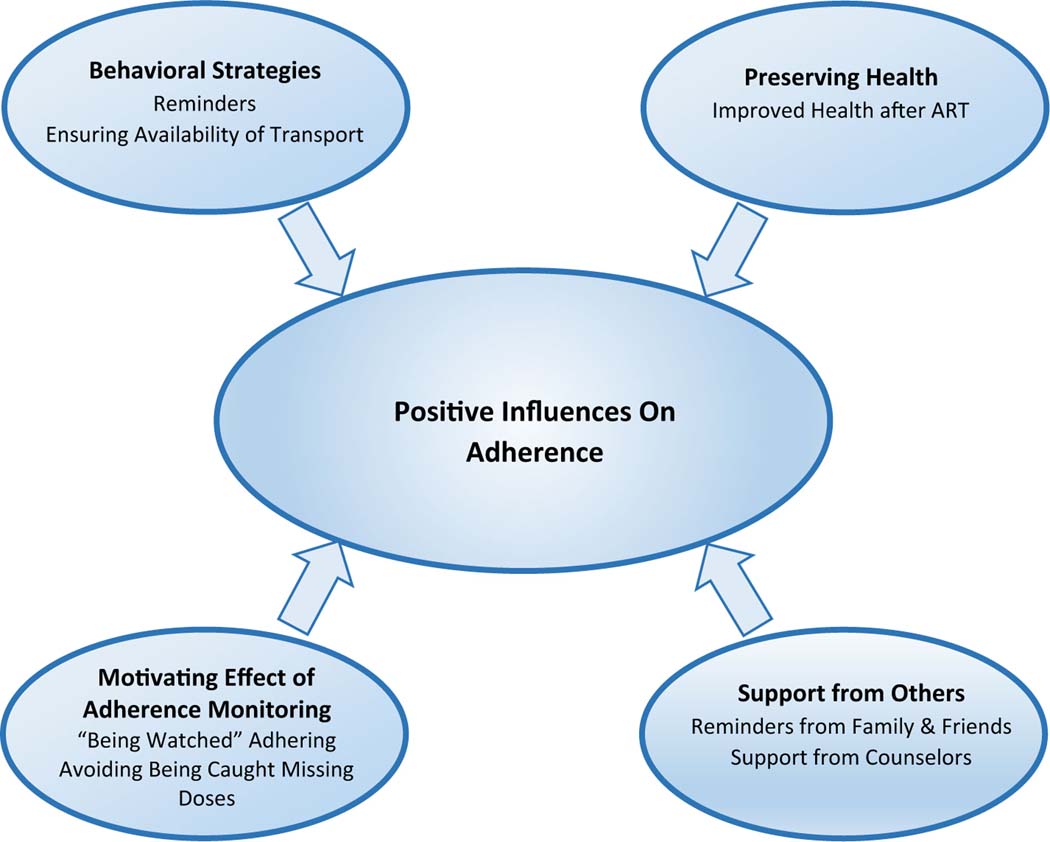
Schematic representation of positive influences on ART adherence in early HIV disease identified through qualitative analysis

There was general agreement among interviewees that the monitoring device meant one’s adherence was “being watched.” Being informed by study staff at each follow-up visit of how many doses they had missed since the last appointment corroborated this feeling. Having one’s adherence behavior “watched” clearly motivated adherence, either because being watched was interpreted as “caring” on the part of clinic staff, or because participants preferred to avoid being challenged on missed doses (Table 2, 4) (Fig. 1).

### De‑stabilizing Experiences

The remainder of Results explores the proposition that de-stabilizing experiences represent barriers to adherence. “De-stabilizing experiences” are defined as disruptions to daily routines and life patterns that make adherence to daily doses of medication more difficult. “Mobility”, “Loss” and “Pregnancy” sections below describe three types of de-stabilizing experiences and their consequences that appeared repeatedly in the qualitative data. The final section draws upon the data to describe the negative impact of these consequences on adherence.

#### Mobility

Mobility—voluntary and involuntary—was part of life for many qualitative study participants, who changed residences, left home for extended periods, or were without a permanent residence altogether. Moves were made to improve economic circumstances—to look for opportunities elsewhere, succeed in a job that required travel, or maintain a farm “in the village.” Mobility also accompanied changes in intimate relationships, whose endings and beginnings meant partners moved out or moved in. Some moves were not desired, as women, in particular, could be forced to flee their homes to escape domestic violence. The de-stabilizing impact of mobility lay in the burdens and demands of working to establish a new life in a different location (Table 3, 5).

#### Loss

Loss was also prominent in the experiences of study participants, who reported losing jobs, being robbed of possessions, and frequent deaths of family members and friends. Ending partnered relationships also meant losses. Partnerships ended due to infidelities—confirmed and suspected, unwanted pregnancies, and disclosure of positive HIV status. Losses were de-stabilizing in their emotional impact, and because they exacerbated poverty (Table 3, 6).

#### Pregnancy

Pregnant women in the qualitative sample typically reported being happy about their pregnancy. The experience of being pregnant, however, could be de-stabilizing. Women worried about the impact of HIV on the progress of the pregnancy and the health of the unborn child. They suffered from malaise and physical symptoms they did not fully understand and found demoralizing. Being pregnant sometimes brought on debilitating fatigue that prevented women from working to generate income. In the absence of support from a male partner and/or family members, women became poorer as a result of being pregnant (Table 3, 7).

#### De‑Stabilizing Experiences as Barriers to Adherence

The negative impact of de-stabilizing experiences on adherence appeared in the interview data in a number of ways. Experiences that resulted in impoverishment (loss, pregnancy) led, for some, to food shortages. Food shortages contributed to missed doses for interviewees who feared unpleasant symptoms would ensue from taking ART on an empty stomach. Some pregnant women, already feeling ill, skipped doses rather than risk exacerbating their symptoms as a result of taking ART. Mobility interfered with adherence when interviewees traveled without their pills and returned home later than expected. In some cases, being away from home or moving prevented returning to the clinic at which ART had been initiated for re-supply. Seeking prescription refills from a health facility at which one was not registered could result in being required to repeat the initiation process, or in being turned away (See Table 3, 8).

**Fig. 2:**
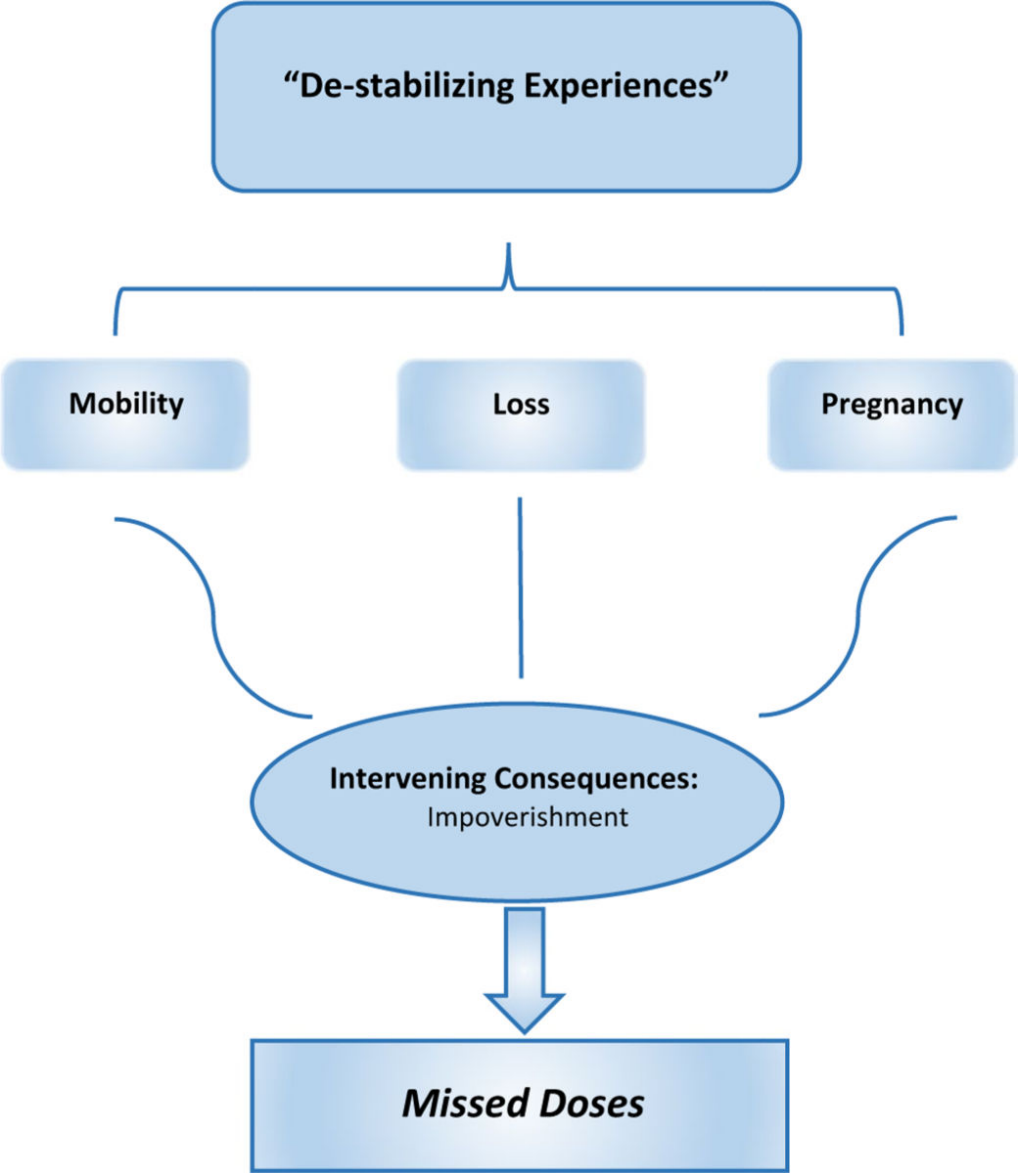
“De-stabilizing experiences” as barriers to adherence

Any one of these de-stabilizing experiences alone could undermine adherence, but typically it was a combination of factors that tipped the balance away from being able to take pills consistently. For example, one pregnant woman ran away from her husband following a physical assault. She had been taking ART for 2 months, and in the confusion, left her medication behind. When she left, she went to her mother’s home some kilometers away, only to discover her mother had moved. As a result, she had no place to stay, and difficulty obtaining food. Without sufficient food and water, the pills seemed to make her ill. As a result, she would decide “to miss the drugs today.” This woman’s loss of her home and resulting mobility was impoverishing, contributing to food shortage and resulting missed doses (Table 3, 8) (Fig. 2).

## Discussion

This qualitative analysis has sought to characterize influences on adherence for adults initiating ART early in HIV disease. Four positive influences and three “de-stabilizing experiences” have been described. Participants readily reported behavioral strategies that helped them take their daily dose of medication at the designated time. The motive to preserve health, support from others, and the experience of adherence monitoring in real time were also cited as positive influences. Interviewees also described ways in which daily routines and patterns were disrupted; the consequences of these disruptions, e.g. the exacerbation of poverty, made adherence more difficult, leading to missed doses. Adherence was high among qualitative study participants overall.

Over the years, social support has emerged as a major influence on ART adherence in sub-Saharan Africa. A recent, comprehensive review of patient-reported barriers and facilitators cited support from family and friends “most reported facilitator” of ART adherence [19]. Participants in this study emphasized the importance of the support they received from counselors they met in the course of HIV testing and initiation of treatment, as well as from friends and family members. Social support may prove an important factor in early stage ART adherence, as well as later in the HIV disease course.

The positive influences on adherence described here have also been cited as adherence facilitators in previous investigations of ART adherence in later-stage HIV disease, despite an overall tendency in earlier research to focus principally on barriers. The use of behavioral strategies has been frequently reported [20–25]. The desire to preserve health has been represented both as a “stand alone” influence and as the result of feeling better after initiating ART [20, 26, 27]. Real-time electronic adherence monitoring is relatively new, but inquiries into patient experiences of monitoring to date point to a strong motivating effect [28]. A desire to maintain the appearance of good health has been identified as a factor prompting HIV testing and early treatment among young South Africans [29]. The appeal of appearing healthy could be persuasive in encouraging early treatment initiators to adhere to ART.

The small, but growing, body of qualitative research examining experiences of ART in the context of “test-and-treat” is relevant here, as test-and-treat necessarily means an increase in the number of persons initiating ART early in HIV disease. Two recent qualitative studies reporting on these experiences make reference to influences on early ART use. Horter et al. [30] draw attention to the importance of seeing a need for treatment despite the absence of symptoms, and of virologic testing to confirm treatment effectiveness. Adams and Zamberia [31], focusing exclusively on eSwatini men, identify social barriers likely to result in delayed ART initiation despite increased availability of ART through test-and-treat. Both investigations adopt a broader analytic approach than in our study, which focuses “close-up” on day-to-day experiences of pill-taking.

Barriers and facilitators of ART adherence among pregnant women have been extensively investigated, and a large number of related factors identified [32–34]. Curiously absent from these investigations, however, is an inquiry into how the subjective experience of pregnancy itself may bear upon ART use. We sought to understand the impact of being pregnant upon adherence by examining the descriptions of pregnancy offered by women in our sample. Their accounts suggest that pregnancy is a de-stabilizing experience that has a meaningful, but indirect impact on the ability to sustain a daily regimen of ART. Interviewees cited certain negative consequences of being pregnant, which in turn negatively affected adherence. For example, as we have seen, pregnancy-associated fatigue interfered with income-generation, which led to food shortage, which led to reluctance to dose, for fear of illness resulting from taking ART on an empty stomach.

Pregnancy is one of three de-stabilizing experiences characterized as adherence barriers on the basis of this analysis. We have proposed that the negative adherence impact of de-stabilizing experiences makes itself felt via intervening consequences, such as the exacerbation of poverty. This proposition is based on a qualitative data analytic strategy that allows for interpretation as well as description of adherence influences reported by study participants. A more interpretive approach is more inclusive, allowing analysts to reach for significant factors that may be outside the awareness of participants—and thus not reportable—but nonetheless very real. While not accessible to participants for reporting in response to open-ended questions about adherence influences, such factors appear in stories of adherence (and life) experiences, suggesting their import.

This study is perhaps the first to qualitatively explore experiences of ART adherence in early-stage HIV disease. We acknowledge several limitations, as follows. Qualitative data collection was limited to a single interview; follow-up interviews with participants would have added depth to the analysis. Social desirability—the tendency to say what one believes one’s listener prefers to hear—may have played a role in shaping interviewee responses. Qualitative research designs prioritize variability in study samples to set the stage for detailed analysis, but they are generally not well-suited to systematic analysis of differences among sample sub-groups. Where subgroup differences were clearly evident in our data, we embedded them in the presentation of categories describing adherence influences, in “Results” section. Finally, as qualitative research, results are not intended to be generalizable.

## Conclusion

Results of this qualitative study point to substantial overlap between factors positively influencing ART adherence in early-stage disease and those identified in previous research as impacting adherence later in the disease course. Similarities in types of influences suggest existing adherence support strategies and interventions—shown to be effective in addressing barriers “later adherers” face—may be helpful in promoting adherence regardless of when persons living with HIV initiate treatment. De-stabilizing experiences and other indirect, but important influences on ART adherence merit additional investigation across varying populations.

## Figures and Tables

**Table 1:** Personal characteristics of qualitative study participants, compared to the remainder of the META study population

	Median (IQR) or N(%) Qualitative Sample (N = 100)	Median (IQR) or N(%) Remainder of Study Population (N = 804)	p value
Age (years)			
Median	27 (24,33)	31 (26, 38)	< 0.001
Mean	30 (10)	33 (10)	0.0027
Gender—Female (N/%)	78 (78)	547 (68)	0.050
Marital status (N/%)			
Single/living alone	47 (47)	330 (42)	0.52
Married/Lvg w/partner	45 (45)	347 (43)	
Separated/divorced/widowed	8 (8)	115 (15)	
Number of living children			
Median	2 (1, 3)	2 (1, 3)	0.14
Mean	2.3 (1.7)	2.6 (4)	0.49
Highest level of education (N/%)			
None/primary only	30 (30)	214 (27)	0.46
Secondary	61 (61)	529 (67)	
Post-secondary	9 (9)	49 (6)	
Employment			
Employed	43 (43)	278 (35)	0.12
Unemployed	57 (57)	514 (65)	
Self-rated health at baseline			
Poor	4 (4)	114 (14)	< 0.001
Fair—good	62 (62)	559 (71)	
Very good—Excellent	34 (34)	118 (15)	
CD4 count (cells/mm^3^): median (IQR) at baseline	446.5 (390, 529)	369 (126, 462)	< 0.001
Log viral load (copies/ml): median, IQR at baseline	4.2 (3.2, 4.7)	4.5 (3.6, 5.1)	< 0.001
Viral suppression (< 50 copies/ml) at month 12 (N/%)	78 (81)	561 (82)	0.89
Adherence (by Wisepill)			
At month 6			
Mean	73 (31)	74 (28)	0.91
Median	88 (61, 98)	86 (59, 96)	0.46
At month 12			
Mean	69 (33)	70 (29)	0.77
Median	81 (49, 95)	80 (50, 94)	0.78

Qualitative study participants have been removed from the META study sample for purposes of this comparative analysis. The total number of participants enrolled in the META Study is 904 [17]

**Table 2:** Interview excerpts illustrating positive influences on adherence Category name Elaboration Illustrative interview excerpts

Category name	Elaboration	Illustrative interview excerpts
1. Behavioral strategies to support adherence	Reminders of dosing time, e.g. alarms, TV/radio programs, Wisepill device	“I have an alarm in my phone. So it goes off and I’d know the time [to take my pills].” Woman, S. Africa, Age 22 “[The Wisepill device] helps me because it is not easy for people to know what is inside, or that I keep my tablets inside. …It also works as reminder that I should get that thing from the cupboard and drink my pills.” Woman, S. Africa, Age 20
	Ensuring transport would be available on clinic appointment days	“When I know I do not have [money for] transport I wake up very early at about 6:00 a.m. and start walking. When the [motorcycle taxis] are many, you can plead with one and say, ‘please drop me there.’ When you reach there you make believe it is where you are going. Then you again get someone else, you again plead with that person to drop you somewhere. You keep doing that till the time you reach the destination. If they had brought you straight you would have to pay because no one can bring you up this side without charging. So you get rides in bits until you reach the clinic.” Man, Uganda, Age 37
2. Preserving health through adherence	Experience of improved health after initiating ART	”My health was not good; I would get fevers, headaches and also feel weak. But when I started these drugs of mine, the headaches and fevers stopped. The drugs have changed everything about my life and made it well.” Woman, Uganda, Age 31 “Now I see that I have taken some time without getting sick from flu or cough. This is what causes me to continue swallowing it because now I feel I have good health. So I have to continue swallowing it, to remain healthy.” Woman, Uganda, Age 23
	Desire to preserve the appearance of good health	“I have seen people who are HIV positive refuse to swallow ART drugs...When I see the way they look I really see that they do not look good. So I say to myself, ‘let me swallow my drugs so that I do not become like so and so.’ I swallow my drugs every day at the same time so that it helps my life.” Woman, Uganda, Age 25
	(Pregnant women) ensuring the health of children	“Since I started treatment, I take my pills each day at 8 o’clock because I have a duty...I have a life to take care of.” Woman, S. Africa, Age 22 “I said, ‘let me take the drugs and swallow them so that I do not infect the child. ‘ I have children, and if you refuse to take it you get sick and become bedridden and your children lack someone to take care of them. So that is what forces me to take my drugs. So that I can continue looking after my children, so that they grow and I also get good health.” Woman, Uganda, Age 33
3. Support from others	Reminders to take pills from family and friends	“Most of the time we [my children and I] are together, [and] they will ask, ‘do you know what the time is? It’s almost 9 pm. We are going to watch [TV program]. What about the tablets?’” Woman, S. Africa, Age 38 “In the morning before I go to work you hear [my wife] say, ‘You have not swallowed your drugs.’ That is the help she gives me. She reminds me about my drugs since she knows my status. At times she reminds me about my clinic date. She says, ‘your dates are near.’” Man, Uganda, Age 31 “My friends whom I work with are the ones who say, ‘We are going to put the things [tools] away...have you got out the drugs?’ So when I have forgotten, they at times remind me. When it’s towards evening you hear them say, ‘But [Name], you have not yet swallowed your drugs.’” Man, Uganda, Age 31
	Support from counselors	“[Counselor told me], ‘you are not the only one. All these people you are seeing here at one point were healthy like you were, but they got infected with the disease. So it’s not that when you get AIDS then you should kill yourself or become something else. You have only to be patient and continue to swallow your drugs properly. ‘” Woman, Uganda, Age 31

**Table 3:** Interview excerpts illustrating de-stabilizing experiences as barriers to adherence

Category name	Elaboration	Illustrative interview excerpts
5. De-stabilizing experience: mobility	Mobility → difficulty establishing oneself in a new location	“I separated with the man that I was staying with.... I had gone to my mother’s place after my husband’s place....[He] denied the pregnancy and said I was lying to him. When I went to my mother’s village, I found my mother had got married elsewhere after separating with my father. My grandmother could not manage to care for me so it was hard for me. I suffered for some time.” Woman, Uganda, Age 24
6. De-stabilizing experience: loss	Loss of relationship with baby’s father → impoverishment	“Since I got pregnant, the relationship is not the same. I mean he is not coming to my house anymore... I told him I wanted to tell him something, it seemed he already knows what I am talking about. He did not come; he did not care. I can see that he is running away from this. I told him I needed to buy baby essentials to come out of hospital. The cash he gave me was not enough to cover all costs. He has not even seen the child.” Woman, S. Africa, Age 29 “Before I was pregnant I would provide everything by myself. When I gave birth. I was not able to provide anything for myself. He [baby’s father] has not brought anything, even soap. If I do not have food I have to go to someone’s field and dig and I get money and then I come and buy maize flour. He has never sent me even 100 shillings to buy some oil.” Woman, Uganda, Age 23
7. De-Stabilizing experience: pregnancy	Pregnancy → malaise, physical symptoms, depressed mood	“I realized that I missed having my menstrual periods on the days I expected to have them. I also started to feel bad and got changes in my life. I started vomiting, getting sick and did not feel fine. ...I did not want to eat anything. I also felt hatred for my life; I did not want to see any one. I lost all my peace.” Woman, Uganda, Age 21
8. De-stabilizing experiences as barriers to adherence	Traveling away from home → missed doses	“One day I had travelled with my cousins to visit my aunts. I left [the pills home] because we were not sure how long we were going to stay there. So it happened that we were still there by the time I should have been taking them. ... And again when we were at the villages (Eastern Cape). It gets really busy there in December, so it got busy. It would come to the time of taking tablets and I am not in the house.” Woman, S. Africa, Age 27
	Mobility → difficulty refilling prescriptions	“I move from one place to another and at times you find that you started drugs long ago, and you have to lie and then you end up testing as a new client. You test as a new person and when you say you are just starting they give you small drugs thinking you are just starting yet you have already reached on the level of taking the big ones. I even went some other place where they refused to give me the drugs. They told me to go back to where I was from and get a transfer letter. When they told me that, I also decided to leave all that and I stopped taking the drugs.” Woman, Uganda, Age 24 “You [can’t] just go to any health facility. You must have a specific center where they monitor you. Now for me at times I may be in [name of district]. You might even go to South Africa for a course. You have to come here first unless you are spending there a short period of time. You have to come here and they give you drugs. If you are to take long they cannot give you all those drugs. It means you have to travel from that place and come here which is a challenge.” Male, Uganda, Age 29
	Pregnancy—impoverishment—food shortage → missed doses	“When I was pregnant it would at times hinder me from going to work. [Then] I would fail to get money to buy things - like porridge - to swallow the drugs with, because I cannot use water. When I didn’t work and get money, I would say, ‘no, let me miss this tablet and not swallow it.’” Woman, Uganda, Age 23 “That used to happen to me when I was pregnant. I knew that if I swallow the drugs when I had not eaten I would not get up, because it would make me dizzy, so I would not swallow. But now that I am not pregnant it is very rare for me to fail to get what to eat.” Woman, Uganda, Age 20 “At times I would be lacking something to eat. When you swallow this medicine without eating it can kill you; it has a lot of power. So when I would see that I do not have food I would say, ‘today I will not swallow it.’” Woman, Uganda, Age 24
	Pregnancy—malaise and physical symptoms → missed doses	“It was not easy for me because I became sick and they started me on drugs [ART], which treated me badly. I then stopped the drugs for some time because I knew that the drugs were the ones that had caused me poor health. I did not know that the pregnancy could also make me feel bad. When they took me to the counselor she told me, ‘Continue taking your drugs; the drugs do not have a problem. Since you are pregnant it may be the pregnancy is causing you bad feeling.’” Woman, Uganda, Age 18
	Pregnancy—physical assault—mobility—increased poverty—food shortage → missed doses	“When I left that man’s place and went to my mother’s village... she had got married elsewhere, so she was not there. So when I reached there, the situation was difficult. When I would get back from digging [to earn income during the day], I would have to start looking for something to prepare. The food was not good, so I would say, ‘I am not going to swallow these drugs for them to kill me.’ I would only swallow if I had something to eat. That’s what used to make me fail to swallow.” Woman, Uganda, Age 24
